# Sclerostin antibody stimulates periodontal regeneration in large alveolar bone defects

**DOI:** 10.1038/s41598-020-73026-y

**Published:** 2020-10-01

**Authors:** Yao Yao, Frederic Kauffmann, Shogo Maekawa, Lea V. Sarment, James V. Sugai, Caroline A. Schmiedeler, Edward J. Doherty, Gill Holdsworth, Paul J. Kostenuik, William V. Giannobile

**Affiliations:** 1 Department of Periodontics and Oral Medicine, University of Michigan School of Dentistry, Ann Arbor, MI 48109 USA; 2 Biointerfaces Institute, University of Michigan, Ann Arbor, MI 48109-2800 USA; 3 Department of Biomedical Engineering, College of Engineering, University of Michigan, Ann Arbor, MI 48019 USA; 4 Department of Oral and Craniomaxillofacial Surgery, Center for Dental Medicine, University Medical Center Freiburg, 79110 Freiburg, Germany; 5 Department of Periodontology, Graduate School of Medical and Dental Sciences, Tokyo Medical and Dental University, Tokyo, 113-8510 Japan; 6 Wyss Institute for Biologically Inspired Engineering at Harvard University, Cambridge, MA 02115 USA; 7 UCB Pharma, Slough, SL1 3WE UK; 8 Present Address: Department of Oral Medicine, Infection, and Immunity, Harvard School of Dental Medicine, Boston, MA 02115 USA

**Keywords:** Regenerative medicine, Periodontitis

## Abstract

Destruction of the alveolar bone in the jaws can occur due to periodontitis, trauma or following tumor resection. Common reconstructive therapy can include the use of bone grafts with limited predictability and efficacy. Romosozumab, approved by the FDA in 2019, is a humanized sclerostin-neutralizing antibody (Scl-Ab) indicated in postmenopausal women with osteoporosis at high risk for fracture. Preclinical models show that Scl-Ab administration preserves bone volume during periodontal disease, repairs bone defects surrounding dental implants, and reverses alveolar bone loss following extraction socket remodeling. To date, there are no studies evaluating Scl-Ab to repair osseous defects around teeth or to identify the efficacy of locally-delivered Scl-Ab for targeted drug delivery. In this investigation, the use of systemically-delivered versus low dose locally-delivered Scl-Ab via poly(lactic-co-glycolic) acid (PLGA) microspheres (MSs) was compared at experimentally-created alveolar bone defects in rats. Systemic Scl-Ab administration improved bone regeneration and tended to increase cementogenesis measured by histology and microcomputed tomography, while Scl-Ab delivered by MSs did not result in enhancements in bone or cemental repair compared to MSs alone or control. In conclusion, systemic administration of Scl-Ab promotes bone and cemental regeneration while local, low dose delivery did not heal periodontal osseous defects in this study.

## Introduction

Alveolar bone defects often result from periodontal disease, trauma or tumor resection^1–3^, leading to tooth loss, esthetic limitations, and/or other periodontal issues, such as challenges with precision dental implant placement or fixation. Reconstruction of large alveolar bone defects around teeth remains clinically problematic because it requires new bone formation within a large lesion area, as well as cementogenesis, concomitant with periodontal ligament fiber reattachment^4,5^. In general, reconstructive therapies of alveolar bone often involve autologous or allogeneic bone grafts along with guided bone regeneration (GBR)^6^, which can be invasive and may cause potential surgical complications with limited predictability and efficacy^7^. To address this need, bone-forming therapies that can increase bone volume and improve bone quality in a more predictable and less invasive manner would be beneficial for periodontal regenerative medicine. Wnt signaling is a key mediator of bone formation, and agents that promote Wnt signaling may be promising alternatives or adjuvant therapies to repair bony defects^8^.

Sclerostin is a glycoprotein secreted primarily by osteocytes that acts as a negative regulator of bone formation by inhibiting canonical Wnt signaling^9,10^. Sclerostin inhibition leads to increased canonical Wnt signaling in bone and increased bone formation^11–13^. The effect of sclerostin inhibition was further inferred from patients with life-long sclerostin deficiency, such as sclerosteosis or van Buchem disease, who exhibit increased bone mass^14,15^. Given that previous basic studies and naturally occurring diseases show the important role of sclerostin in osteogenesis, the pharmacological inhibition of sclerostin by a neutralizing antibody has been tested across various clinical trials and preclinical models of bone loss^16^. Clinical trials in women with postmenopausal osteoporosis (PMO) show that 12 months of treatment with Evenity (romosozumab-aqqg), a humanized anti-sclerostin antibody (Scl-Ab), reduced the risk of osteoporotic fractures compared with placebo^17^ and compared with the bisphosphonate alendronate^18^. Based on those studies, Evenity was approved in 2019 by the Food and Drug Administration (FDA) and other regulatory agencies as a bone-forming treatment for women with PMO at high risk of fracture^19^.

Sclerostin antibodies have also been investigated preclinically for other bone loss and bone injury settings. Scl-Ab administration led to enhanced bone formation in a critical-sized femoral defect model in rats^20^, and Scl-Ab administration also increased bone volume, bone mineral density, and alveolar bone height after experimental periodontitis^21^. Moreover, Scl-Ab reversed alveolar bone loss in a rat model of chronic edentulism^22^ and Scl-Ab also improved mechanical fixation of oral implants by enhancing regeneration of the supporting bone^23^. Such results indicate that Scl-Ab may have utility as a bone anabolic agent for treating large alveolar bone defects by increasing bone volume and improving bone quality.

The systemic delivery of Scl-Ab is an attractive therapeutic option because the efficacy of systemic Scl-Ab administration to treat bone loss has been demonstrated across various studies^24^ and is administered to patients once per month to achieve satisfactory outcomes^25^. During the past decade, in order to achieve high administration effectiveness of proteins or peptides, numerous biomaterial studies have shown that novel polymeric particulate carriers, such as biodegradable PLGA microspheres (MSs), can be an effective way to locally control drug release and be easily adapted to complex defects in a less-invasive manner compared to conventional surgery^26^. As an FDA-cleared biomaterial, PLGA microspheres have served the healthcare sector by contributing many commercial products to combat various diseases, including Eligard to treat prostate cancer, Zoladex to treat breast cancer, Arestin to treat periodontitis, and Nutropin to treat growth hormone deficiency^27,28^. Using a large alveolar bone defect model, this study compared the efficiency of locally delivered versus systemic administration of Scl-Ab as a bone anabolic agent to treat a large alveolar osseous defect around teeth to stimulate periodontal regeneration. Bone and cementum regeneration were then analyzed by microcomputed tomography (µCT), histology, and histomorphometry.

## Results

### Synthesis and characterization of microspheres

**Figure 1:**
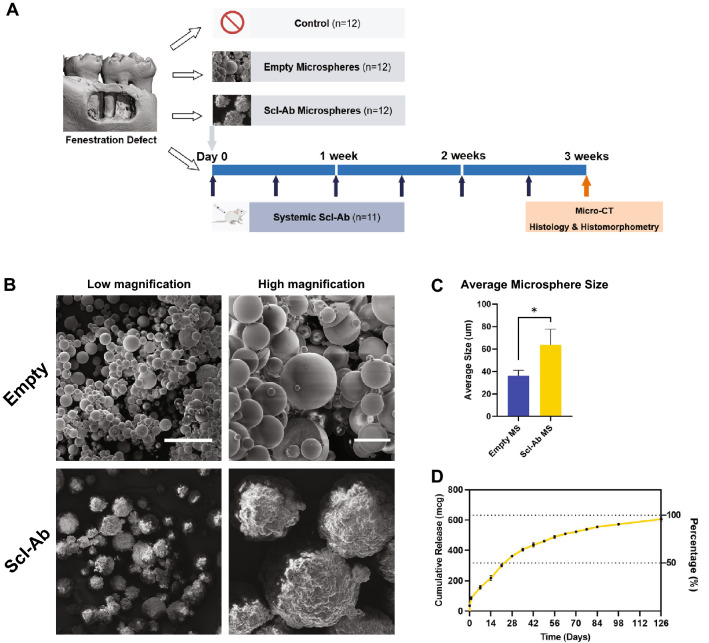
(**A**) Study design and timeline of the in vivo experiment; (**B**) Scanning electron microscopy images of the empty and sclerostin antibody (Scl-Ab) PLGA microspheres (MSs) at low and high magnifications. Scale bars indicate 100 μm (left) and 20 μm (right); (**C**) Mean size (± SD) of empty MS and Scl-Ab MS (empty MS: n = 3; Scl-Ab MS: n = 4); (**D**) 18-week time course of Scl-Ab release from PLGA MS at 37 °C. * = p < 0. 05.

The empty PLGA MSs and Scl-Ab loaded PLGA MSs were successfully obtained by a water-in-oil-in water emulsion solvent evaporation method. The size difference between the loaded and unloaded microspheres is related to the flow dynamics of the solutions with or without the protein being present (Fig. 1C). The mean size (± SD) of Scl-Ab MSs and empty MSs were 63.75 ± 14.03 μm and 36.33 ± 4.73 μm, respectively. Both MSs had spherical morphologies as evidenced by scanning electron microscope (SEM), but somewhat differing surface topography due to the presence of Scl-Ab on the outer surface and high vacuum utilized during the SEM (Fig. 1B). Figure 1D illustrates the cumulative in vitro 18-week Scl-Ab release profile from the PLGA MSs. Around 20% of Scl-Ab was initially released during the first 24 h, followed by an 18-week long sustained in vitro release in phosphate-buffered saline (PBS) as estimated by BCA assay. In the in vitro release model approximately 50% of the Scl-Ab was released from the microspheres within 3 weeks, while another 50% was released in a much slower manner.

### Large alveolar bone defect model

**Figure 2:**
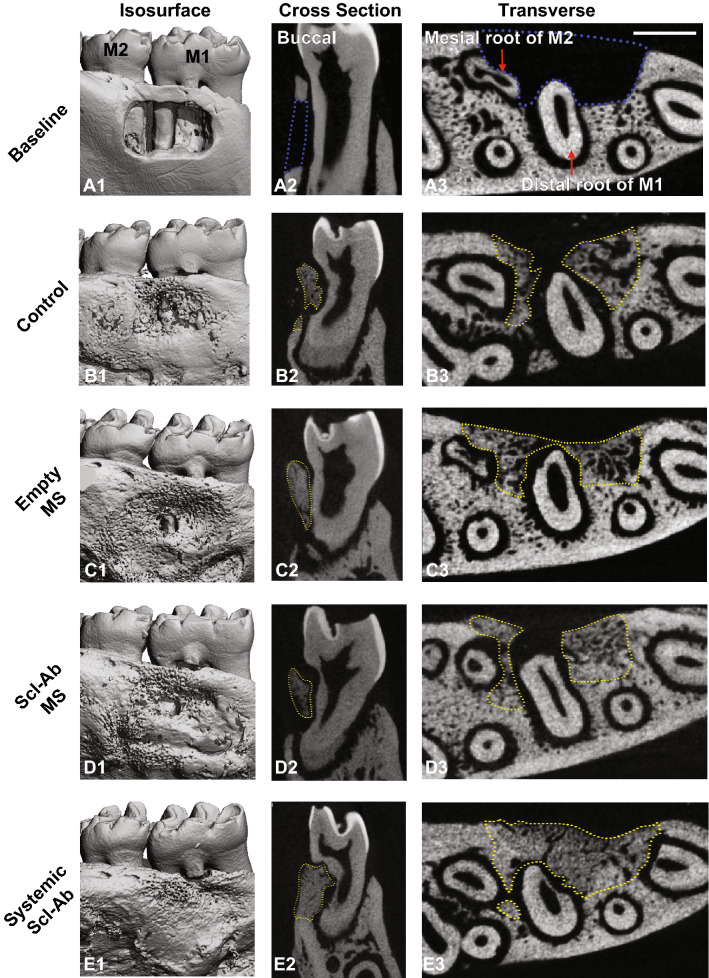
Representative μCT images of the fenestration defect exposing the distal root of first molar (M1) and the mesial root of second molar (M2) at (**A**) baseline (bone defect areas are indicated by blue lines), and (**B**–**E**) 3-week time points within various treatment groups (control, empty MS, Scl-Ab MS: n = 12; systemic Scl-Ab: n = 11). 3D isosurface images (**A1**–**E1**), 2D cross-sectional (**A2**–**E2**), and transverse views (**A3**–**E3**) highlight the visual differences between the area and density of bone regenerated within the defect (indicated by yellow lines). Scale bar indicates 1 mm.

Figure 2 A1-A3 shows representative micro-CT reconstructions of the large periodontal fenestration defect from a rat mandible immediately after surgery. The defect had a standardized size of ~ 3 mm × 2 mm × 1 mm, which was located on the buccal side of the mandible (Fig. 2 A1-A2) as described previously^29^. Transverse view images (Fig. 2 A3) confirmed that the distal root of the first molar was exposed in the mid-portion of the defect, and the mesial root of the second molar was also exposed posteriorly.

From our histological analysis, the edge of the bone defect, which is an obvious interface between original bone and new bone, can be identified as the indicator to define the linear length between edge to edge of bony defect as “defect length”. Terminal bone histomorphometry showed that mean (± SE) bone defect length in the control, empty MS, Scl-Ab MS, and systemic Scl-Ab groups was 2.62 ± 0.08, 2.77 ± 0.08, 2.70 ± 0.04, and 2.77 ± 0.08 mm, respectively (all *p* > 0.05). Mean bone defect area in the control, Empty MS, Scl-Ab MS and systemic Scl-Ab groups was 1.73 ± 0.09, 1.92 ± 0.07, 1.81 ± 0.06, and 1.69 ± 0.08 mm^2^, respectively (all *p* > 0.05). There were no significant differences in lengths and areas of the experimental bone defects, demonstrating consistent surgical technique among all groups (Table 1).

**Table 1:** Histomorphometric analysis at 21 days after surgery.

		Control	Empty MS	Scl-Ab MS	Systemic Scl-Ab
Length of new bone (mm)		2.52 ± 0.07	2.60 ± 0.12	2.43 ± 0.14	2.69 ± 0.09
Linear bridging bone (%)		96.4 ± 2.0	93.8 ± 3.4	89.9 ± 4.8	97.3 ± 1.8
Area of new bone (mm^2^)		0.92 ± 0.07	0.89 ± 0.06	0.83 ± 0.09	1.03 ± 0.05
Bone fill %		53.9 ± 3.5	46.0 ± 2.8	45.3 ± 4.1	61.2 ± 3.0^a,b^
New cementum	+	6/12	6/12	7/12	4/9
New cementum length (mm)		0.39 ± 0.08	0.40 ± 0.09	0.38 ± 0.10	0.58 ± 0.12
New cementum length (%)		32.8 ± 7.1	31.9 ± 7.5	32.4 ± 8.5	52.5 ± 9.8
Root resorption	+	3/12	7/12	6/12	1/9

For lengths and areas of new cementum/root resorption.

a; p < 0.05 compared to empty MS.

b; p < 0.05 compared to Scl-Ab MS.

Tukey–Kramer test.

For positive numbers of New Cementum and Root Resorption.

No significant differences.

Chi-square test.

### Microcomputed tomographic (μCT) analysis of regenerated tissue

**Figure 3:**
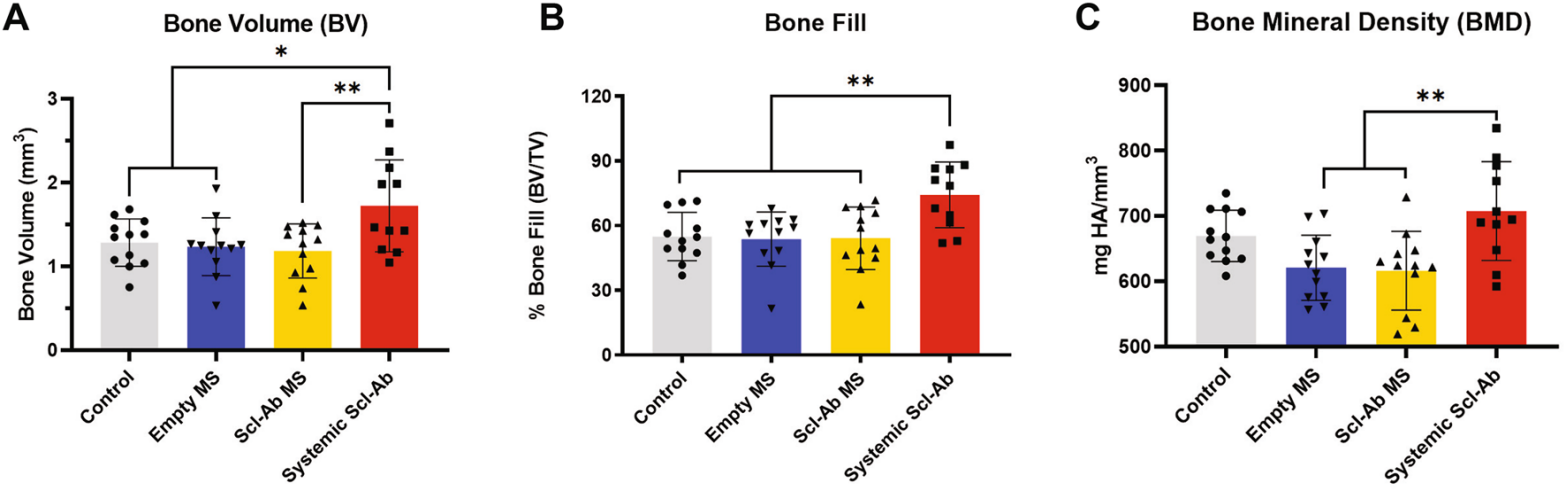
Microcomputed tomography (μCT) assessments of bone volume (**A**), bone fill (**B**), and bone mineral density (**C**) at 21 days after surgery (control, empty MS, Scl-Ab MS: n = 12; systemic Scl-Ab: n = 11). Systemic Scl-Ab group showed significant differences for bone volume, bone fill and bone mineral density compared to both MS groups by ANOVA and Tukey’s post hoc test. No difference was found between localized deliveries (empty MS or Scl-Ab MS). * = p < 0. 05, ** = p < 0.01.

Quantitative μCT measurements showed that bone volume and bone fill in the systemic Scl-Ab group was approximately 40% greater than the other three groups (Fig. 3A,B). The bone mineral density of the systemically treated Scl-Ab group was also significantly higher than empty MS and Scl-Ab MS groups (Fig. 3C).

Based on μCT measurements, representative images were chosen and shown in Fig. 2. Control, empty MS, and Scl-Ab MS groups achieved partial bone repair by day 21, leaving some residual bone defects on the buccal surface (Fig. 2B–D). Compared with these groups, near-total bone fill, which bridged the entire lesion area, was seen more routinely in the systemic Scl-Ab group (Fig. 2E1), indicating increased bone bridging and osteogenesis. As bone with varying densities could be recognized based on different grayscale levels, the newly-formed bone in the systemic Scl-Ab group displayed greater density and was less distinguishable from the surrounding native bone (Fig. 2E3), suggesting a higher bone density as compared to the other groups^30^.

### Histological and histomorphometric analysis of regenerated tissue

**Figure 4:**
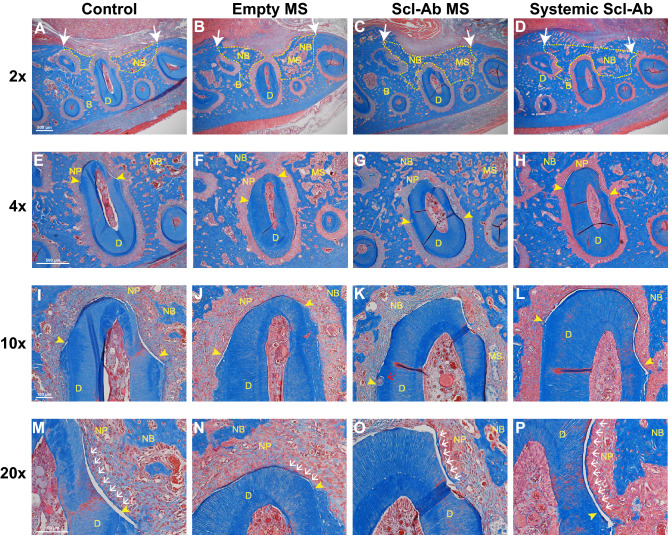
Histological analysis of periodontal healing at 21 days after surgery. The sections were stained with Masson’s Trichrome. The ×2 images of alveolar bone defect and newly formed bone areas are shown (**A**–**D**). White arrow indicates the edges of the original bone defect and newly bone areas are outlined with dashed yellow line. 4 × images of the distal root of the first molar (**E**–**H**). Yellow arrowheads demarcate the edges of exposed tooth dentin surface. 10 × images of newly formed periodontal tissue at the distal root of the first molar (**I**–**L**). 20 × images for newly formed cementum-like tissues (M-P). Scale bars indicate 500 μm (**A**–**H**) and 100 μm (**I**–**P**). D, dentin; B, bone; NB, new bone; MS, microspheres; NP, newly regenerated periodontal ligament. Yellow arrowheads, the edge of exposed tooth dentin; white arrows, new cementum; red arrows, root resorption; Sample sizes were 12 in Control, 12 in empty MS, 12 in Scl-Ab MS and 9 in the systemic Scl-Ab group.

Images of the bone defect and newly formed bone areas are shown in Fig. 4A–D. Blinded histological analyses indicated that two specimens showed surgical pulp exposure and destruction of dentin and were therefore excluded from assessment. After excluding the specimens, these two were de-identified to belong to systemic Scl-Ab group. The final sample size was 12 each in the control, empty MS, and Scl-Ab MS group, and 9 in the systemic Scl-Ab group.

At day 21, many specimens showed bone regeneration along with bridging among the groups, leading to no significant differences in new bone length. However, the newly formed bone area fill (%) in the systemic Scl-Ab group was significantly greater compared to both the empty MS and Scl-Ab MS groups (Table 1). High magnification images around the distal root of the first molar are shown in Fig. 4E–H. The mean lengths of exposed dentin in the control, empty MS, Scl-Ab MS and systemic Scl-Ab groups were 1.22 ± 0.08, 1.24 ± 0.06, 1.21 ± 0.09, and 1.10 ± 0.03 mm, respectively. There were no significant differences in lengths of exposed tooth dentin.

The newly formed cementum is shown in both low power images (Fig. 4 I–L) and in high power images (Fig. 4M–P). Newly formed cementum was observed in some but not all specimens 21 days after surgery. New cementum length (%; mean ± SE) was 52.5 ± 9.8% in the systemic Scl-Ab group and less than 33% in the other groups (shown in the Table 1), with a trend for higher values in the systemic Scl-Ab group versus control. Interestingly, some microspheres were associated with isolated fibrous tissue either scattering around the bone surface or aggregation near the periodontal ligament (Fig. 4G,K). Root resorption was found in some specimens (Fig. 4K) but less in the systemic Scl-Ab group, and there was no significant difference (p = 0.09, data not shown).

## Discussion

The findings from this investigation demonstrate that systemically-administered Scl-Ab was associated with improved osseous repair of large alveolar bone defects in comparison with the effects of local Scl-Ab formulated in PLGA microspheres, microspheres alone, or no treatment. These findings build on the large body of evidence demonstrating that systemic Scl-Ab therapy stimulates new bone formation and increased alveolar bone volume and density following experimental periodontitis and around large bone defects at the time of dental implant placement^21,23,31^. These results suggest therapeutic potential for this approach in a variety of dental applications. The systemic Scl-Ab group showed significant enhancement of alveolar bone healing after surgery including bone volume, bone fill, and bone mineral density based on μCT analysis. These findings are also consistent with the previous reports about enhancing vertebral structural bone parameters using Scl-Ab in osteoporosis models^32^, and alveolar bone in a molar extraction model^22^. Moreover, histomorphometric analysis confirmed that the percentage area of new bone fill was significantly greater by systemic delivery of Scl-Ab versus control, as well as a tendency for increased cementogenesis and less root resorption. Taken together with our previous study using an experimental periodontitis model^21^, systemic Scl-Ab therapy can enhance periodontal regeneration in different dental conditions of alveolar bone loss around teeth.

As a new bone-forming therapy, Scl-Ab and its underlying mechanisms on bone formation have generated significant attention over the past decade. At the tissue-level, Scl-Ab has been demonstrated to initially augment bone volume by increasing modeling-based bone formation, which occurs directly on quiescent bony surfaces^33^. Furthermore, the sustained indirect anti-resorptive effect also contributes to the overall increase in bone. At the cellular level, the administration of Scl-Ab increases bone formation by converting quiescent lining cells, which arise from osteoblasts covering 94% of bone surface, into active osteoblasts^34^ as well as increasing osteoprogenitors and osteoblast vigor^30,35^.

Our study employed two different delivery methods to administer Scl-Ab: (i) local delivery by PLGA MSs at bone defect surfaces and (ii) systemic delivery by subcutaneous (s.c.) injection, but the results of bone formation exhibited significantly differences between local and systemic groups. There are several possible mechanisms for the outcome. One potential mechanism that may have limited the efficacy of Scl-Ab MSs on bone regeneration induced by PLGA biodegradation in vivo in osseous defects. The tissue response to biodegradable microspheres can be characterized as occurring in three phases^36^. Phase I occurs within the first two weeks following implantation including the initiation of the acute and chronic inflammatory responses, which is generally similar regardless of the degradation rate. Phase II of the tissue response to biodegradable polymers is initiated by the predominance of monocytes and macrophages and the length of the second phase in dependent on the degradation rate of the microspheres. Visscher et al. shown that 50:50 PLGA microspheres have a phase II response of 50 to 60 days^37^. Phase III is characterized by the breakdown into particles of microspheres, which is also dependent on the degradation rate, which may have influenced drug release kinetics and delayed bone healing starting at early time points^38^. Our results also showed some isolated fibrous-like tissues associated with microspheres (Fig. 4F,G). As indicated previously that inflammatory response may not resolve until the disappearance of the polymer fragments^39^, a rapid degradation rate may enable microspheres more suitable for periodontal regeneration. In addition to the degradation rate, the acidic by-products during PLGA biodegradation also lead to local inflammation when implanted in vivo. Considering both aspects of degradation rates and degradation by-products, the continued investigation of alternative materials such as gelatin or modified PLGA microspheres^40^ with more rapid degradation rates and harmless by-products upon degradation^41^ that elicit less local inflammation are promising drug-delivery devices for future study^38,42^.

Another potential mechanism could attribute to the different dosing levels between local and systemic groups. The loading limit of PLGA MSs with Scl-Ab was maximized by the formulation process with 125 µg per defect, however still significantly much lower as compared to systemic administration (31.25 mg in total per animal). Although strategies to increase the incorporation of Scl-Ab in MSs may develop in the future, the possibility of providing local delivery with Scl-Ab dosing as high as systemic administration remains unlikely because the small dimensions of these defects (~ 6mm^3^) would not allow for the administration of 31.25 mg of Scl-Ab even without PLGA or other carriers. Although numerous advantages of local drug delivery from implanted polymers have been demonstrated during the past decade, including reduction of unwanted systemic side effects, elimination of repeated administration, and increased patient compliance^43,44^, significant limitations of drug-loaded polymers still exists, particularly for therapeutic antibodies that are much larger and therefore occupy far more space than small molecules or peptides. These limitations include insufficient loading capacity, unsatisfied polymer biodegradation, and material-mediated inflammation.

One limitation of the current study is the lack of a vehicle control group to account for possible non-specific effects of the twice weekly s.c. injection protocol. Another limitation is the different dose levels administered locally versus systemically. The high dose used systemically was as a positive control based on doses used in previous alveolar bone models, and it is likely that lesser efficacy would have occurred had the systemic dose matched the local dose. The third limitation is the lack of dynamic histomorphometric analysis of tissue or bone remodeling events occurring during early wound healing.

Considering the unique characteristics of Scl-Ab for the promotion of bone density in a variety of contexts, several studies have reported the ability of Scl-Ab to address unmet dental needs. Scl-Ab administration has previously shown inhibition of periodontal disease progression^32^, enhancement of the recovery from periodontal disease^21^, and increased osteointegration and bone regeneration around dental implants^23^. Moreover, in two large clinical studies, a single case of osteonecrosis of the jaw (ONJ) bone was reported^17,18,31,32^ whereas antiresorptive agents including bisphosphonates and denosumab are associated with an increased risk of ONJ. Though further research is needed for dental applications, systemic administration of Scl-Ab may have potential as an alternative or adjuvant therapy for various clinical oral applications such as blocking periodontal tissue progression, regeneration of periodontal and peri-implant defects, and enhancing the volume and density of alveolar bone. If Scl-Ab is shown to increase alveolar bone formation and bone density in humans, it could be envisioned that patients with postmenopausal osteoporosis may choose to coordinate the timing of their Scl-Ab therapy with dental reconstruction procedures as a way of leveraging alveolar bone responses to Scl-Ab. In conclusion, our study showed systemic administration of Scl-Ab enhances bone regeneration and may stimulate new cemental formation in large alveolar defects above that of local, low dose delivery of Scl-Ab using biodegradable PLGA microspheres. The use of Scl-Ab offers significant potential for the promotion of alveolar bone regeneration for future application in human alveolar bone reconstructive strategies.

## Materials and methods

### Animals, large alveolar bone defect surgery, and treatments

All animal procedures were performed with approval from the University of Michigan Institutional Animal Care and Use Committee according to the ARRIVE guidelines for preclinical studies. 48 Sprague Dawley male rats (6-week-old, weight ≈ 250 g) (Charles River Laboratories, MA) were acclimatized, then administered preemptive s.c. analgesic (Carprofen, 5 mg/kg body weight). The four surgical treatment groups were fully randomized among all 48 rats to ensure the surgeon and assistant were blinded to remove any bias. One rat with evident pulp exposure during the surgery was excluded from the study and analysis. The final number of rats that underwent the surgical procedure was 47, including 12 rats for control, 12 for empty MS, 12 for Scl-Ab MS, and 11 for systemic Scl-Ab group.

Under general anesthesia with isoflurane, a 15–20 mm horizontal superficial incision was made on the buccal side of the right mandible followed by separation of the skin tissue and the muscle layer^45^. A second incision was carefully made through the muscle to expose the alveolar bone. All the surgeries were conducted using a surgical microscope as previously described^29,43^. A single fenestration defect was created using round surgical burs with decreasing diameter by exposing the distal root of the first molar tooth and the mesial root of the second molar tooth and removing the buccal root of the first molar tooth. The surrounding bone and cementum layer were carefully removed to expose the dentin surface. The standardized dimensions of the defect in the buccal area were ~ 1 mm deep and ~ 3 mm mesiodistally^29^. During the surgeries, the surgeon ensured that the crestal bone coronal to the defect maintained its integrity and was approximately 1 mm to prevent communication with the oral cavity. Defects were then treated either with Scl-Ab MS (n = 12), or empty MS (n = 12), or nothing (n = 23). The total amount of MS per defect was 2.5 mg containing 125 µg Scl-Ab or empty MS, which was suspended in 25 μL of PBS by a technician while keeping the surgeon blinded. Twenty five microliters of MS suspension were then carefully pipetted into the defect using a P100 pipettor and were given approximately 30 s to fully set into the defect. A multilayer soft tissue wound closure was subsequently performed to secure the intact periosteum above the defect and contain the MS. During recovery, each animal was maintained on its left side to promote the loaded material to stay in position. Another four animal with an untreated defect was sacrificed immediately after surgery by CO_2_ overdose to serve as a baseline control for micro-CT. The site was closed using resorbable sutures (muscle layer) and surgical staples, with s.c. administration of analgesic (carprofen, 5 mg/kg) 24 h post-surgery. The following 48 h after surgery the animals were given a 5% glucose water supplemented with ampicillin (268 mg/l) to prevent potential infection. At the same day of surgery, one group of rats with untreated defects received systemic s.c. injections of Scl-Ab (25 mg/kg) twice weekly for 3 weeks (n = 11), while the remaining animals with untreated defects remained untreated (n = 12) (Fig. 1A). All animals were euthanized 3 weeks post-surgery by CO_2_ overdose, and mandibles including first, second, and third molars were collected, carefully dissected and fixed in 10% buffered formalin phosphate solution for 3 days before being transferred into 70% ethanol for subsequent analyses.

### Microsphere preparation

Poly (lactic-co-glycolic acid) (PLGA) microspheres were fabricated using a water in oil in water (w/o/w) emulsion solvent evaporation method. 50 mg of sclerostin antibody (Scl-Ab; r13C7): the preclinical research equivalent of romosozumab, which contains the unchanged complementarity determining regions from romosozumab, with frameworks and constant regions including the Fc region, substituted from the rat to reduce immunogenicity in rodents (UCB Pharma/Amgen Inc.) was added to 4 ml of dichloromethane (Sigma-Aldrich, USA) solution containing 450 mg of GMP grade 50:50 PLGA (Durect Lacte, AL, USA) and then emulsified using a probe sonicator to form a water/oil emulsion. The water/oil suspension was added to 2% (w/v) solution of polyvinyl alcohol (PVA) (Sigma-Aldrich, MO, USA) and emulsified using a homogenizer for 2 min at 5000 rpm. The resultant emulsion was added to 500 ml of 0.25% (w/v) PVA solution and mechanically stirred for 4–5 h to allow evaporation of the dichloromethane and solidification of the microspheres. The microspheres were then centrifuged at 5000 rpm for 15 min to separate for PVA solution. This process was repeated with water for injection twice more by re-suspending the pellet in water and centrifuging. Microspheres were then frozen, lyophilized and stored at -20 °C.

### Microsphere characterization

Particle size of the PLGA microspheres was evaluated by a laser diffraction particles size analyzer (Malvern Instruments, UK). The amount of Scl-Ab loading and release was estimated in PBS at room temperature using a Micro BCA protein assay kit (Thermo Scientific, MA, USA). Microspheres were sputter-coated with gold for 120 s (SPI-Module Carbon/Sputter Coater) and observed under a scanning electron microscope (Tescan RISE) at 2 kV.

### Microcomputed tomography (µCT) analysis

Microcomputed tomography (µCT, Scanco Medical, Switzerland) was used to evaluate bone regeneration post-surgery. In order to make the micro-CT analysis consistent and reliable, we determined a Hounsfield (HU) = 1080 for bone, set all µCT parameters to the same grayscale and brightness level to ensure no difference during the entire analysis. All 47 mandibular specimens were embedded in alginate, scanned using a µCT system at a resolution of 12 μm, 90 kV energy, and 155 μA intensity, and calibrated to HU (Hounsfield Units) = 1080 for bone. Scans were reconstructed and 2D (cross section and transverse planes) and 3D isosurface images were generated for all specimens. Using Scanco software (SCANCO Medical AG, Switzerland), each reconstructed image was rotated into a standardized orientation. A masked examiner performed all measurements using the same monitor and first measured the height of every single defect individually and selected the middle plane as 0. The volume measured was 0.5 mm above and below (apically and coronally) from the middle plane. For every single slice, researchers drew the contours of newly-formed bone areas as shown in Fig. 2B3,C3,D3,E3 and the whole defect areas as shown in Fig. 2A3 based on visible indicators such as the cementum removed during the surgery and the differences in density of native bone and newly-formed bone as previously described with minor modifications^46^. A total of 84 slices with 12 µm thickness each were analyzed, which led to around 1 mm (84 * 12 = 1.008 mm) height in total. The bone volume (BV) and total volume (TV) were than calculated and generated by the software (Scanco). According to the instructions from the manufacturer, bone mineral density (BMD) was calculated based on the mean voxel values of everything within volume of interest in a specific setting for bone tissue. In brief, the scanning was calibrated for bone, then the mean voxel value obtained from data analysis is in units of hydroxyapatite density [mg HA/ccm].

### Histology and histomorphometry

After scanning, samples were decalcified in 10% EDTA, at 4 °C for 4 weeks, then embedded in paraffin, and cut into 5 μm transverse sections for histological analysis. Some sections were stained with Masson’s Trichrome for histomorphometric analysis of the bone defect, including newly formed bone and new cementum to the tooth root. Using 2 × magnification sections, we performed bone measurement analyses. First, we identified the edges of the bone defect. Then, we made a straight line connecting the edges, measured the length, and defined this as “the length of the defect” as previously described^43^. When we recognized non-bridging newly formed bone, we made the marks on the edges of the non-bridging area, drew two perpendicular lines on an existing straight line of the length of the defect, and measured the length of non-bridging bone. This non-bridging bone length was subtracted from the length of the defect, and defined as “the length of new bone”. We calculated the linear bridging bone % by the length of newly linear bridging bone length divided by the length of the defect. The area of bone defect was identified by the interfaces between the original bone and newly formed bone outlining the whole defect area. The area of new bone fill (%) was obtained by calculating the ratio between newly formed bone area and bone defect area. Using 10 × magnification sections, we measured the length of new cementum. First, we marked the edges of the exposed dentin at the distal root of the 1st molar and marked along the line of exposed dentin. Then we measured the lengths of exposed dentin. The newly formed cementum was measured in the same manner, then new cementum length % was also calculated by the length of newly formed cementum divided the length of exposed dentin. Root resorption was evaluated using with 10 × and 20 × images. Light microscopy of Trichrome-stained sections was performed using a Nikon Eclipse E800 microscope (Nikon Inc., Melville, NY, USA) and images were captured with a SPOT-2 camera (Diagnostics Instruments, Inc., Sterling Heights, MI, USA) for histomorphometric analysis using NIS-Elements software version BR-3.2 (Nikon Instruments, Melville, NY, USA). To minimize errors, a single examiner (S. M.) was confirmed with high intra-rater reliability (0.96 ± 0.06) and high inter-rater reliability (0.86 ± 0.16) compared to the standard examiner (L. S.). All measurements for histomorphometry were performed on the coded specimens in a blinded fashion. Quantitative analyses were performed using Image J software (v. 1.52q; National Institutes of Health, MD, USA) for linear length measurements, Adobe Photoshop CC 2020 software (Adobe, CA, USA) for specific area measurements (i.e., new bone matrix area) and Adobe Illustrator CC 2020 software (Adobe, CA, USA) for curved line measurements (i.e., lengths of removed cementum and new cementum).

### Statistical analysis

µCT and histomorphometry data were analyzed using Prism 8 software (GraphPad Software, CA, USA). Comparison of the two types of MSs was performed with student’s t-test, while comparisons among multiple groups were done with one-way analysis of variance (ANOVA) followed by Tukey. For the intra-rater and the inter-rater reliability tests and Chi-square test, SPSS statistic software was used to calculate (version 25.0.0.0., SPSS, Inc., IL, USA). A value of p < 0.05 was considered statistically significant.
